# Visualization of strain distribution in rubber bulk during friction

**DOI:** 10.1038/s41598-024-64271-6

**Published:** 2024-06-12

**Authors:** Toshiaki Nishi, Kyohei Ueno, Tomohiro Nomoto, Shinya Sugisawa, Daiki Shin, Ken Yamaguchi, Isao Kuwayama, Takeshi Yamaguchi

**Affiliations:** 1 Graduate School of Engineering, Tohoku University, 6-6-01, Aoba, Aramaki-Aza, Aoba-ku, Sendai, Miyagi 980-8579 Japan; 2 Bridgestone Corporation, Tokyo, Japan; 3 Graduate School of Biomedical Engineering, Tohoku University, Sendai, Japan

**Keywords:** Polymers, Rheology, Imaging techniques

## Abstract

This study employed a digital image correlation method (DICM) to experimentally quantify horizontal strain distribution in silicone rubber bulk during horizontal displacement against a stainless-steel sphere with/without glycerol. The strain distribution at different depth levels was measured by capturing the position of white powders in transparent rubber bulk. The experimental results indicated that each point in the rubber bulk moved while describing a horizontal loop during horizontal displacement depending on the position and lubrication conditions. This caused changes in the horizontal strain during horizontal displacement. These results suggest that the hysteresis term could be caused by changes in the vertical and horizontal strains.

## Introduction

Slip resistance performance is important in products such as vehicle tires and shoes to prevent traffic and slip-and-fall accidents^1–9^. The higher the friction coefficient (*μ*), the lower the risk of such accidents^4–7^. Industry experts and academics have attempted to improve the slip resistance to no avail. The friction force (*F*) is mainly the sum of the adhesion term (*F*_ad_) and hysteresis term (*F*_hys_)^10–12^. The treads of most vehicle tires and outer shoe soles are made of rubber (one of viscoelastic materials) whose *F*_hys_ can be high during horizontal displacement as the softness of rubber increases the strain^11,13^. Considering the road/floor condition during practical use of vehicle tires and shoes, a rough surface of asphalt is the most common, and the *F*_ads_ and *F*_hys_ should be dominant to determine *F*^14,15^. However, lubricant invasion may decrease the contribution of *F*_ad_ to *F* owing to a decrease in the real contact area. Thus, it is reasonable to design the *F*_hys_ to achieve the high slip resistance of vehicle tires and shoes even on wet roads or floors.

It has been reported that an asperity contacting a rubber surface induces vertical and cyclic deformation during horizontal displacement^14^. Considering that rubber is not elastic but a viscoelastic body, the stress in a compression process exceeds that in a recovery process. The stress difference in these processes leads to the *F*_hys_^14^. In an experimental investigation^16^, a master curve of *μ* based on the Williams–Landel–Ferry theory^17^ showed that the viscoelastic properties of rubber could determine *µ*. The relationship between friction behavior and viscoelastic properties has been theoretically investigated based on a combined series of Kelvin elements^18,19^. Furthermore, the strain distribution during rubber friction has been investigated using the finite element method (FEM)^20–25^. However, for common rubber that contains fillers, its viscoelastic properties are determined by the temperature, strain rate, and strain range^26,27^. Thus, it is technically difficult to theoretically estimate the strain distribution with high accuracy. The change in rubber surfaces during horizontal displacement has been experimentally investigated by X-ray imaging. However, research has yet to achieve the measurement of strain distribution in rubber bulk during horizontal displacement^28^. Thus, an experimental method to visualize the strain distribution in rubber bulk during horizontal displacement is required.

The digital image correlation method (DICM) is commonly employed to measure the strain distribution on material surfaces in tensile, tear, and bending tests by tracking random dots on the specimen^29–34^. To the best of our knowledge, no studies have measured the strain distribution in rubber bulk during horizontal displacement using the DICM. By preparing a transparent rubber sheet with a layer of random dots in the rubber, it is expected that the horizontal strain distribution in rubber bulk can be calculated by capturing the change in the position of the dots during horizontal displacement. This study clarified the sub-surface horizontal strain distribution in rubber during sliding. Rubber sheets with a layer of random white dots were prepared by changing the vertical level of the dot layer to investigate the strain distribution at depths from the rubber surface. The friction test was conducted between a rubber sheet and stainless-steel sphere with/without glycerol to investigate the effect of lubrication on strain distribution in rubber bulk.

## Results and discussion

### Friction coefficient

**Figure 1:**
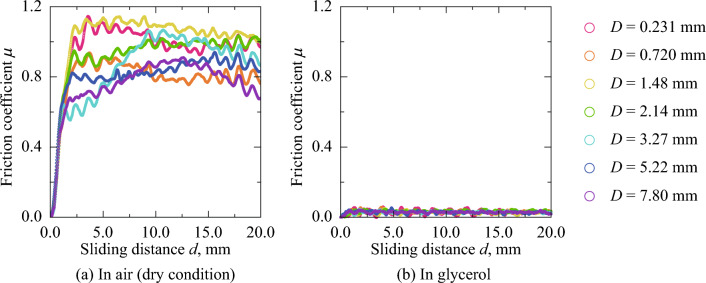
Friction coefficient versus sliding distance. The different colors indicate the results for seven types of rubber sheets where *D* is within the range of 0.231–7.80 mm.

Figure 1 shows the friction coefficient (*μ*) without/with glycerol plotted against the sliding distance, *d*. The different colors indicate the results for seven types of rubber sheets where *D* is within the range of 0.231–7.80 mm. Regardless of the lubricant conditions, *μ* increased with *d* at *d* = 0.0–2.0 mm with a constant value at *d* = 2.0–20.0 mm. Using the results at *d* = 10.0 mm as the steady state, the *μ* of the unlubricated condition (mean *μ* = 0.940, standard deviation = 0.102) was 30.1 times that of the lubricated condition (mean *μ* = 0.0306, standard deviation = 0.0043). Considering that the loss tangent of the silicone rubber was so minimal (~ 0.05), the *F*_hys_ was sufficiently small and the difference in *μ* corresponded to the change in the *F*_ad_. Thus, owing to the high viscosity of glycerol compared with that of air, it was considered that the real contact area between the rubber and steel sphere drastically decreased because of the lubricant invasion.

### Major and minor principal strains during rubber friction against steel sphere

**Figure 2:**
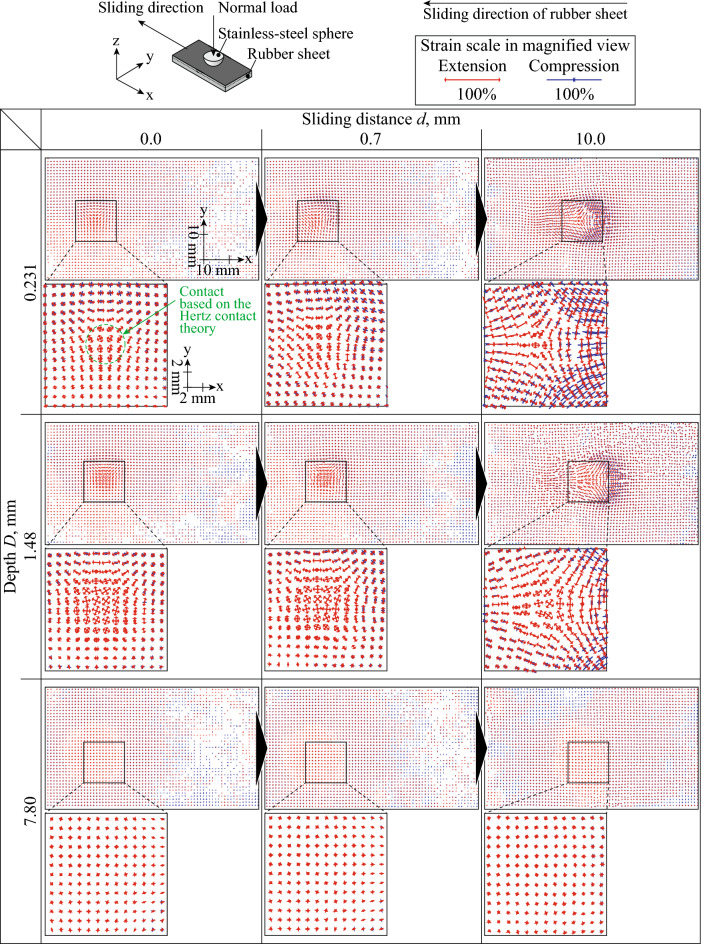
Subset position and vectors of major and minor principal strains in rubber bulk during horizontal displacement against steel sphere at *d* = 0.0, 0.7, and 10.0 mm and *D* = 0.231, 1.48, and 7.80 mm under unlubricated conditions. The red and blue vectors indicate tension and compression, respectively. The cross points of the vectors are the center of each subset. The changes in the position of the subsets were emphasized three times in the *x*- and *y*-directions. Green dashed-line circle indicates the contact calculated based on the Hertzian contact theory.

Figure 2 shows the vectors of the major and minor principal strains at *d* = 0.0, 0.7, and 10.0 mm and *D* = 0.231, 1.48, and 7.80 mm under unlubricated conditions. The red and blue vectors indicate tension and compression, respectively. The cross points of the vectors are the center of each subset. The changes in the position of the subsets were emphasized three times in the *x*- and *y*-directions. Green dash-line circle indicates the outer edge of contact based on the Hertzian contact theory. Regarding the results at *d* = 0.0 mm, which corresponded to the phase of after contacting but before sliding, it was confirmed that, in certain subsets, the major and minor principal strains were positive. At *D* = 0.231 and 1.48 mm, the major and minor principal strains were relatively large at one spot. This spot would be the position under the contact point between the rubber sheet and steel sphere because compression in the *z*-direction could have caused the increase in the major and minor principal strains. The area where both the major and minor principal strains at *d* = 0.0 mm and *D* = 0.231 mm were positive almost corresponded to the contact area calculated based on the Hertzian contact theory. This result explains that the distribution of measured strain would be reasonable at least for the phase before sliding. Interestingly, the major and minor principal strains in this spot at *d* = 1.48 mm exceeded those at *d* = 0.231 mm. The phase at *d* = 0.7 mm corresponded to the phase when sliding was about to occur. Although the strain distribution at *D* = 7.80 mm did not drastically change, the strain distribution at *D* = 0.231 and 1.48 mm exhibited asymmetry in the *x*-direction. This dependency was more prominent at *d* = 10.0 mm. In front of the contact point, the subsets were compressed and extended in the *x*- and *y*-directions, and the positions of the subsets shifted backward. However, the subsets at the rear of the contact point extended in the *x*-direction, with a small strain in the *y*-direction. At this point, the subsets were about to return to their initial position. Owing to a high *μ* for the unlubricated condition, it was considered that the changes in the strain and subset position at *D* = 0.231 and 1.48 mm were caused by *F*. In addition, it can be said that the influence of *F* on the strain distribution and subset position was relatively low at *D* = 7.80 mm.

**Figure 3:**
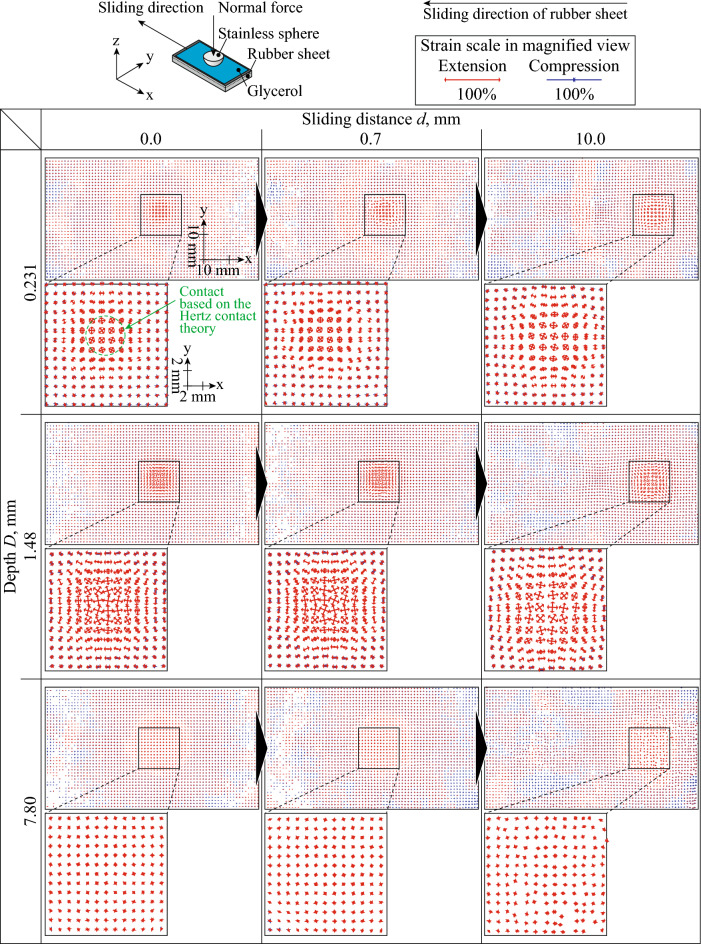
Subset position and vectors of major and minor principal strains in rubber bulk during horizontal displacement against steel sphere at *d* = 0.0, 0.7, and 10.0 mm and *D* = 0.231, 1.48, and 7.80 mm under glycerol-lubricated conditions. The red and blue vectors indicate tension and compression, respectively. The cross points of the vectors are the center of each subset. The changes in the position of the subsets were emphasized three times in the *x*- and *y*-directions. Green dashed-line circle indicates the contact calculated based on the Hertzian contact theory.

Figure 3 shows the position of the subsets and vectors of the major and minor principal strains in rubber bulk during horizontal displacement against the steel sphere at *d* = 0.0, 0.7, and 10.0 mm and *D* = 0.231, 1.48, and 7.80 mm under glycerol-lubricated conditions. Similar to Fig. 2, the change in the subset position is emphasized three times, and the red and blue vectors indicate tension and compression, respectively. The changes in the subset position and strain distribution under glycerol-lubricated conditions were smaller than those under unlubricated conditions. The distribution of the subset position and strain was symmetric during horizontal displacement compared with those under unlubricated conditions. *μ* was considered too small to have affected the distribution.

### Position changes of points in rubber bulk during horizontal displacement

Under unlubricated conditions, the experimental results showed that each subset shifted during horizontal displacement, at least horizontally. To discuss the position change, this section describes the position trajectory. The strain distribution analysis was applied at *d* = 0.0–10.0 mm; therefore, the position trajectory of each subset was practically within 10 mm in the *x*-direction. However, the stress field in the *x*-direction can exceed the analysis range. Thus, the position trajectory was calculated based on the superposition of the position change of five subsets in the same *x*-position.

**Figure 4:**
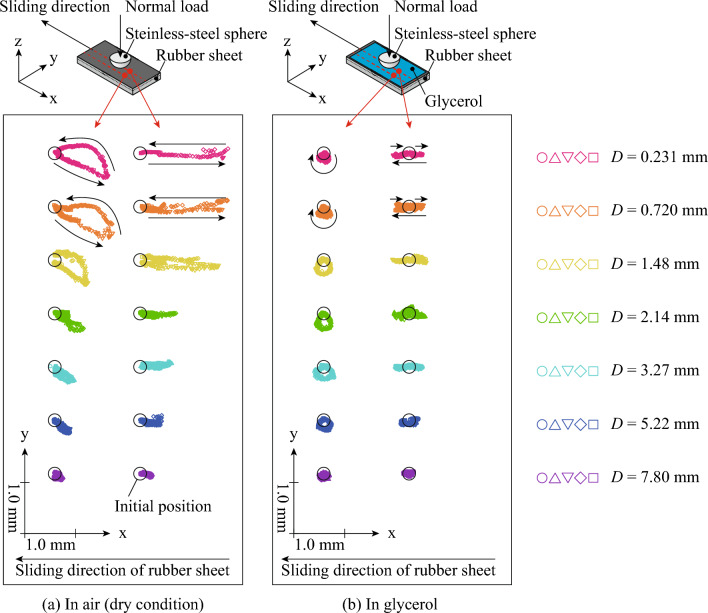
Trajectory of points under and beside the center line of the contact point at *D* = 0.231–0.780 mm under unlubricated and glycerol-lubricated conditions. The subset, 6.0 mm, beside (corresponding to five subsets) the *x*-axis was collected as a result of points beside the center line. (**a**) In air (dry condition), (**b**) In glycerol.

Figure 4a shows the position trajectory of points beside and including the contact point center (center line,* x*-axis) at *D* = 0.231–0.780 mm under unlubricated conditions. The subset, 6.0 mm, beside (corresponding to five subsets) the *x*-axis was collected as a result of points beside the center line. Figure 4a confirms that the points under the center line shifted backward to the sliding direction and returned to the initial position and that the shifting distance decreased with *D*. For the points beside the center line, the position change in the *x*-direction was smaller but similar to that of the points under the center line. However, the points beside the center line shifted to the outer side and returned to the initial position, resulting in a semicircle. An asperity contacting on a rubber surface has been reported to induce vertical and cyclic deformation during horizontal displacement^14^. However, the present study findings experimentally showed that horizontal and cyclic deformation also occurred during rubber friction when contacting a steel sphere.

Figure 4b shows the position trajectory of points under and beside the center line at *D* = 0.231–0.780 mm under glycerol-lubricated conditions. Compared with the results obtained under unlubricated conditions, the position trajectory for glycerol-lubricated conditions was symmetric in the *x*- and *y*-directions, and the shifting distance was smaller. Focusing on the displacement in the *x*-direction, each position shifted backward, forward, and then backward to the sliding direction, resulting in a circle for the points beside the center line. Interestingly, the rotational direction was opposite that under lubricated conditions.

### Strain changes of points in rubber bulk during horizontal displacement

**Figure 5:**
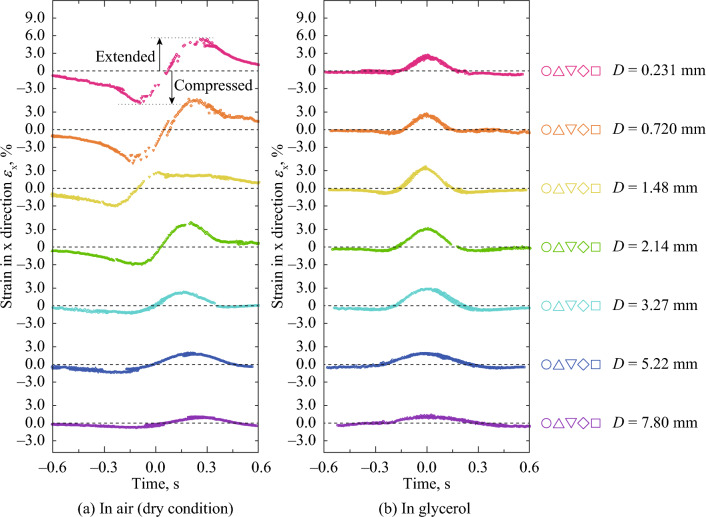
Time dependency of strain in the *x*-direction, *ε*_x_, on the points beside the center line at *D* = 0.231–7.80 mm under unlubricated and glycerol-lubricated conditions; *ε*_x_ > 0 and *ε*_x_ < 0 indicate tension and compression, respectively. Time *t* = 0.0 s corresponded to when the steel sphere passed the subset position. (**a**) In air (dry condition), (**b**) In glycerol.

Apart from the position trajectory, the time dependency of the strain in the *x*- and *y*-directions and the shear strain in the *xy* plane was calculated based on the superposition of the position change of five subsets on the same *x*-axis. It was technically difficult to collect the subsets exactly beneath the center line because the subsets were set at every 1.2 mm. Thus, this section describes the strain change in the rubber bulk beside the contact line. Figure 5 shows the time dependency of strain in the *x*-direction, *ε*_x_, on the points beside the center line at *D* = 0.231–7.80 mm under unlubricated and glycerol-lubricated conditions. Time *t* = 0.0 s corresponded to when the steel sphere passed the subset position. The *ε*_x_ decreased and increased under unlubricated conditions, indicating that the rubber bulk was compressed and extended by *F* during horizontal displacement. The change range in *ε*_x_ was observed to decrease with *D*. Under glycerol-lubricated conditions, the changes in *ε*_x_ were symmetric and the *ε*_x_ peak decreased with *D*. The rubber bulk was slightly compressed before and after passing beside the contact point, whereas the rubber bulk was extended in passing beside the contact point. These results suggested that the strain under glycerol-lubricated conditions was mainly caused by the normal load owing to a small *F* (*μ* = 0.0306).

**Figure 6:**
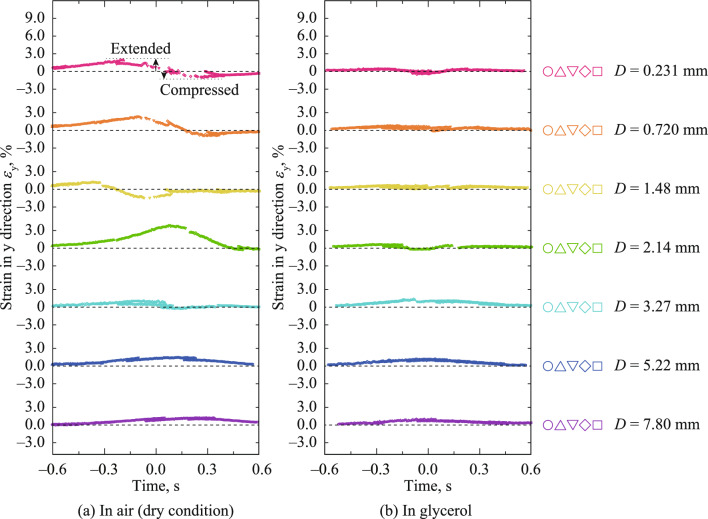
Time dependency of strain in the *y*-direction on the points beside the center line at *D* = 0.231–7.80 mm under unlubricated and glycerol-lubricated conditions; *ε*_y_ > 0 and *ε*_y_ < 0 indicate tension and compression, respectively. Time *t* = 0.0 s corresponded to when the steel sphere passed the subset position. (**a**) In air (dry condition), (**b**) In glycerol.

Figure 6 shows the time dependency of strain in the *y*-direction, *ε*_y_, on the points beside the center line at *D* = 0.231–7.80 mm under unlubricated and glycerol-lubricated conditions. The changes in *ε*_y_ at *D* = 0.231–3.27 mm (unlubricated) and *D* = 0.231–2.14 (glycerol) were complex, resulting in a semicircle or circle. Even if the strain distribution in the *z*-direction was not measured here, the vertical strain could have also caused this behavior. Under the other aforementioned conditions, the rubber bulk was slightly extended in passing beside the contact point, which could have been caused by *F*.

**Figure 7:**
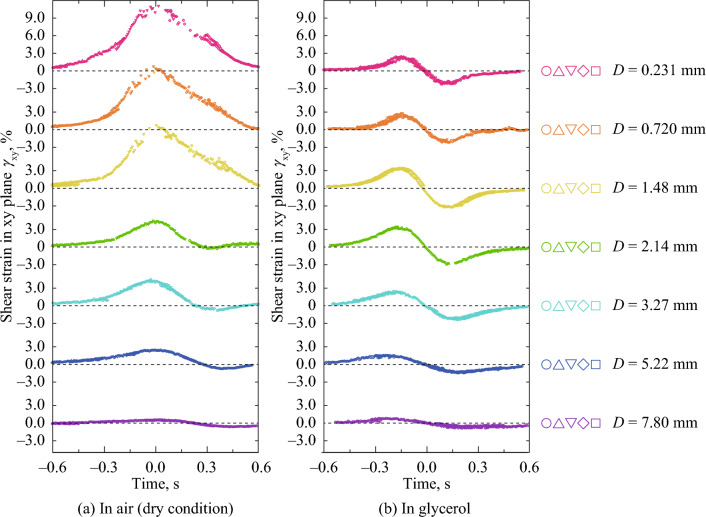
Time dependency of shear strain in the xy plane on the points beside the center line at *D* = 0.231–7.80 mm under unlubricated and glycerol-lubricated conditions. Time *t* = 0.0 s corresponded to when the steel sphere passed the subset position. (**a**) In air (dry condition), (**b**) In glycerol.

Figure 7 shows the time dependency of shear strain in the xy plane, *γ*_xy_, on the points beside the center line at *D* = 0.231–7.80 mm under unlubricated and glycerol-lubricated conditions. In passing beside the contact point, *γ*_xy_ increased, and the maximum values of *γ*_xy_ decreased with *D* under unlubricated conditions. These results were considered to have been caused by *F*. Conversely, under glycerol-lubricated conditions, the *γ*_xy_ was positive and negative before and after passing beside the contact point, respectively. These results could have been caused by the squeezing out of the rubber bulk under the contact point. Contact mechanics has been eagerly studied for decades mainly based on theoretical deductions and numerical simulations^35^. Based on a numerical simulation, Mulvihill et al. investigated the local tangential force within a contact for friction between two hemispheres^36^. It has been reported that the local tangential force if *μ*  > 0 increased and decreased during passing the contact point, and that the local tangential force if *μ* = 0 was positive and negative before and after passing the contact point^36^. Considering the local strain is proportion to the local tangential force, the dependency of *μ* in *γ*_xy_ in Fig. 6 corresponds to the dependency of *μ* on the local tangential force in the previous study, which indicates reasonability of measured strain in this study.

Owing to a low loss tangent (0.0398–0.0717), the *F*_hys_ was small, and the *F*_ad_ was dominant in the rubber friction in the present study. The experimental results indicated that the increase in *F*_ad_ caused an increase in the strain range, resulting in a semicircle-shaped displacement. If the loss tangent was sufficiently large, the *F*_hys_ would increase with the strain range, which can be enlarged by *F*_ad_. Because the loss tangent of rubber for outer shoe soles and vehicle tire treads can be > 0.5, the relationship between friction behavior and strain distribution for rubbers with high loss tangents should be investigated.

## Conclusions

To clarify the horizontal strain distribution in rubber bulk in friction, rubber sheets with a random white dot layer were prepared by changing the vertical level of the dot layer. A friction test between a rubber sheet and stainless-steel sphere with/without glycerol was conducted. The horizontal strain distribution in the rubber sheets at each white random dot layer was measured through the DICM by setting a white random dot layer in a transparent rubber sheet.

*μ* was observed to drastically decrease with glycerol as a lubricant. The DICM results showed that the strain range decreased with *D* (vertical distance from the rubber surface). Using glycerol, the position trajectory of the points in the rubber bulk changed depending on the position in the rubber and the lubricant conditions. It was confirmed that the position trajectory can be a semicircle or circle in passing beside the contact point. This suggests that an asperity contacting a rubber surface induces vertical and horizontal cyclic deformation during horizontal displacement. The proposed methods provide insight into the clarification of the frictional mechanisms of viscoelastic materials, such as rubbers.

## Method

### Rubber sheet specimen

**Figure 8:**
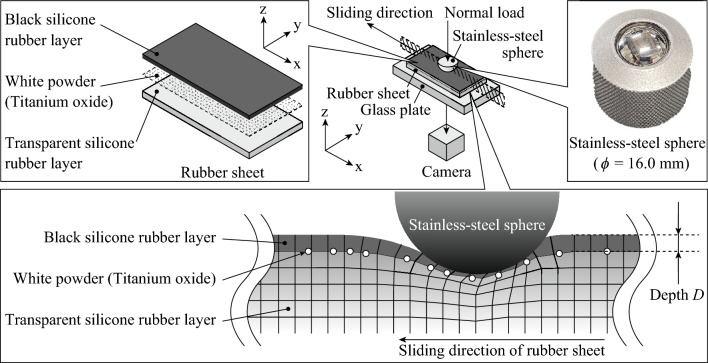
Schematic representation of friction test of rubber sheet specimen and stainless-steel sphere. To measure the sub-surface horizontal strain distribution in rubber during sliding against a steel sphere through the DICM, seven types of rubber sheets with a white random dot layer and black layer were prepared by changing the vertical position of the white random dot layer.

Figure 8 shows the schematic representation of a rubber sheet and stainless-steel sphere (JIS SUS304, AMATSUJI STEEL BALL MFG. CO., LTD., *R*_a_ = 0.56 μm). To measure the sub-surface horizontal strain distribution in rubber during sliding against a steel sphere through the DICM, seven types of rubber sheets with a white random dot layer and black layer were prepared by changing the vertical position of the white random dot layer. The black layer enabled the observation of the white random dots by preventing a backside glare. The depth (*D*) was defined as the vertical depth between a rubber upper surface (surface in contact with a steel sphere) and the white random dot layer. Each rubber sheet was prepared in three steps. First, polydimethylsiloxane (Sylgard 184, Dow Corning Toray Co., Ltd., Tokyo, Japan) containing cure and coloring agents (15 wt% each; food coloring black, Kyoritsu Foods Inc., Tokyo, Japan) was poured into a Petri dish. Thereafter, it was cured at 40 °C for 1 day after vacuuming for a few seconds. Second, a minute amount of the white powder of titanium oxide (TITANE A-190, Sakai Chemical Industry Co., Ltd., Osaka, Japan) was splashed on the black silicone rubber layer through a mesh to form a random dot layer. Finally, the polydimethylsiloxane containing a 15 wt% cure agent was poured on the random dot layer and cured at 40 °C for 1 day after vacuuming for a few seconds. *D* was measured by observing the cross-section of each rubber sheet using a One-Shot three-dimensional (3D) measuring macroscope (VR3000, Keyence Corporation, Osaka, Japan). It was set to 0.231, 0.720, 1.48, 2.14, 3.27, 5.22, and 7.80 mm. The total thickness of the rubber sheet was 10.0 mm. The storage modulus was measured using a rheometer (ARES-G2, TA Instruments, Inc., Guyancourt, France). The G* of the black and transparent silicone rubbers were 1.05 and 0.881 MPa, respectively, at a temperature, oscillation strain, and frequency of 25.0 °C, 0.100%, and 1.00 Hz, respectively. The loss tangent for the black and transparent silicone rubbers were 0.0717 and 0.0398, respectively, under the same conditions, which shows that the silicone rubber was practically an elastic body. The arithmetical mean height (*S*_a_) was measured from a 1.000 mm-square area on the rubber sheets and steel sphere using the One-Shot 3D measuring macroscope. For the steel sphere, plane correction was applied in the accompanying software. The *S*_a_ range of the rubber sheets with different *D* values was 0.461–0.814; that of the steel sphere was 7.414 μm.

### Friction test

Figure 8 shows the schematic representation of the friction test. Each rubber sheet was fixed on a glass plate (BK7; width, length, and thickness of 130, 300, and 20 mm, respectively) using two clamps (SHC-200W, Fujiwara Sangyo Co., Ltd., Hyogo, Japan). The steel sphere (*ϕ* = 16.0 mm) was set on the rubber sheet with a normal load (9.81 N, dead weight). The rubber sheet on the glass plate was linearly moved at 20.0 mm/s for 20.0 mm using a linear stage driven by a servo-motor guided with a ball screw (73K0098, SHINTO Scientific Co., Ltd., Tokyo, Japan). *F* was measured using a load cell (TL201, Trinity Lab., Inc., Japan) at 1,000 Hz. The random dot layer was observed through the glass plate using a camera (HAS-L1, DITECT Corporation, Tokyo, Japan) at 150 fps before the steel sphere contacted the rubber sheet. The friction test was conducted on each rubber sheet without/with glycerol as a lubricant (Wako 1st Grade, FUJIFILM Wako Pure Chemical Corporation, Osaka, Japan). The atmosphere temperature and relative humidity (RH) were 26 °C and 68%RH, respectively.

### Measurement of horizontal strain distribution

**Figure 9:**
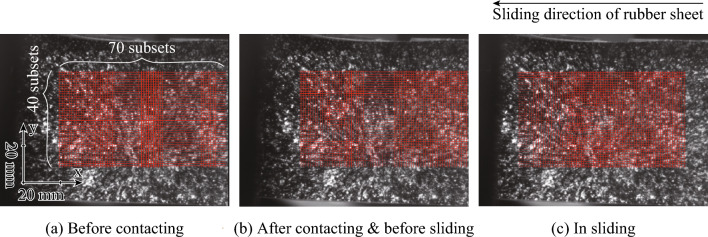
Observed images and subsets using digital image correlation method for friction test without glycerol. 2.4 mm-square areas were defined as subsets on the first observed image (before the contacting phase). The number of lines (*x*-direction) and rows (*y*-direction) of the subsets were set to 70 and 40, respectively, resulting in 2,800 subsets. The interval in the *x*- and *y*-directions was set to 1.2 mm. Each subset was indicated as a red square. (**a**) Before contacting, (**b**) After contacting & before sliding (**c**) In sliding.

The strain distribution during the friction test was quantified from the observed images through the DICM using software (DIPP-Strain, DITECT Corporation, Tokyo, Japan). Figure 9 shows the observed images before and after contact, as well as before and during the sliding phase. Here, 2.4 mm-square areas were defined as subsets on the first observed image (before the contacting phase). The number of lines (*x*-direction) and rows (*y*-direction) of the subsets were set to 70 and 40, respectively, resulting in 2,800 subsets. The interval in the *x*- and *y*-directions was set to 1.2 mm. Each subset was indicated as a red square. The change in position of each subset during horizontal displacement was detected using the intensity distribution^37^. Here, the intensities at *x* = *X* and *y* = *Y* in continuing frames were defined as *I*_n_(*X*, *Y*) and *I*_n–1_(*X*, *Y*), respectively. Defining *u* and *v* as the position shifts of the subsets in the *x*- and *y*-directions, respectively, the total difference in intensity in each subset *C*(*X* + *u*, *Y* + *v*) was defined in Eq. (1).

1 $$\begin{array}{c}C\left(X+u,Y+v\right)=\sum_{i=-M}^{M}\sum_{j=-M}^{M}\left\{{I}_{n}\left(X+u+i,Y+v+j\right)-{I}_{n-1}\left(X+i,Y+j\right)\right\}.\end{array}$$

By minimizing *C*(*X* + *u*, *Y* + *v*), *u* and *v* were determined in each subset for each frame. Using the values of *u* and *v*, the major principal strain, minor principal strain, strain in *x*-direction (*ε*_x_), strain in *y*-direction (*ε*_y_), and shear strain along the *xy* plane (γ_xy_) were calculated until *d* reached 10.0 mm.

## Data Availability

The datasets used and/or analysed during the current study available from the corresponding author on reasonable request.
